# Mesenchymal Stem Cell-Derived Extracellular Vesicles to the Rescue of Renal Injury

**DOI:** 10.3390/ijms22126596

**Published:** 2021-06-20

**Authors:** Lucy Birtwistle, Xin-Ming Chen, Carol Pollock

**Affiliations:** 1 Faculty of Medicine and Health, Sydney Medical School, University of Sydney, Camperdown, NSW 2050, Australia; lbir3487@uni.sydney.edu.au; 2 Kolling Institute, Sydney Medical School, University of Sydney, Royal North Shore Hospital, St Leonards, NSW 2065, Australia; xin-ming.chen@sydney.edu.au

**Keywords:** extracellular vesicle, exosome, mesenchymal stem cells, acute kidney injury, chronic kidney disease, miRNA

## Abstract

Acute kidney injury (AKI) and chronic kidney disease (CKD) are rising in global prevalence and cause significant morbidity for patients. Current treatments are limited to slowing instead of stabilising or reversing disease progression. In this review, we describe mesenchymal stem cells (MSCs) and their constituents, extracellular vesicles (EVs) as being a novel therapeutic for CKD. MSC-derived EVs (MSC-EVs) are membrane-enclosed particles, including exosomes, which carry genetic information that mimics the phenotype of their cell of origin. MSC-EVs deliver their cargo of mRNA, miRNA, cytokines, and growth factors to target cells as a form of paracrine communication. This genetically reprograms pathophysiological pathways, which are upregulated in renal failure. Since the method of exosome preparation significantly affects the quality and function of MSC-exosomes, this review compares the methodologies for isolating exosomes from MSCs and their role in tissue regeneration. More specifically, it summarises the therapeutic efficacy of MSC-EVs in 60 preclinical animal models of AKI and CKD and the cargo of biomolecules they deliver. MSC-EVs promote tubular proliferation and angiogenesis, and inhibit apoptosis, oxidative stress, inflammation, the epithelial-to-mesenchymal transition, and fibrosis, to alleviate AKI and CKD. By reprogramming these pathophysiological pathways, MSC-EVs can slow or even reverse the progression of AKI to CKD, and therefore offer potential to transform clinical practice.

## 1. Pathophysiology of AKI and CKD

Acute kidney injury (AKI) and chronic kidney disease (CKD) are established as global health burdens, with a prevalence of one in ten adults for CKD [1]. AKI refers to a sudden deterioration in renal function, resulting in increased plasma creatinine and blood urea nitrogen (BUN) and/or declining urine output within hours to days [2,3,4]. The most common causes are pre-renal with hypovolaemia, ischaemia, toxic injury, and sepsis, leading to oxidative stress, inflammation, apoptosis, and necrosis of tubular epithelial cells (TECs) [5]. In response, the remaining TECs proliferate and differentiate so recovery occurs over a few weeks [5,6,7].

However, these nephroprotective mechanisms can be overwhelmed and there is a 25% risk of progression to CKD [8], and a 50% increase in 10-year mortality, particularly from coronary events [9,10]. The most common cause of CKD is diabetes, followed by glomerulonephritis, hypertension, and polycystic kidney disease, with smoking, a family history of kidney failure, obesity, ≥60 years old, and being of Aboriginal or Torres Strait Islander origin contributing to the overall risk [1,11]. In Type 2 diabetes mellitus (T2DM), hyperglycaemia induces oxidative stress and inflammation, which leads to maladaptive repair by fibroblasts and podocytes, and reduction of peritubular endothelial capillary (PTC) networks [5,12,13]. A notable mechanism is the epithelial-to-mesenchymal transition (EMT), whereby TECs and fibroblasts transform into myofibroblasts and deposit TGF-β1 and extracellular matrix (ECM) proteins (collagen IV, fibronectin), thereby perpetuating the cycle of glomerulosclerosis and tubulointerstitial fibrosis [5,12,14,15,16]. The fibrotic process strongly correlates with a loss of renal function and CKD is defined by elevated urinary albumin excretion ≥30 mg/g and/or a decrease in estimated glomerular filtration rate (eGFR) to <60 mL/min/1.73 m^2^ for greater than three months [17].

## 2. Current Treatments for Kidney Failure

Current treatments only slow the decline in renal function through lifestyle modifications, managing concomitant cardiovascular disease, or offering dialysis or kidney transplantation [11,18]. Dialysis is the most common reason for hospitalisation [19] and was estimated to cost the Australian government $12 billion from 2009–2020 [20,21]. Quality of life is poor on dialysis [22] and it is often considered a bridge to transplantation as people wait an average of three years on the cadaveric transplant list [23]. Transplantation from deceased or living donors increases five-year survival to 90% and 97%, respectively [24]. However, benefits are limited by donor shortages and the increased incidence of cancer and infections due to long-term immunosuppression [25,26]. Therefore, novel therapeutics slowing the progression of fibrosis and preventing or even reversing the deterioration in renal function are urgently required.

## 3. Regenerative Properties of Mesenchymal Stem Cells

Innovative mesenchymal stem cell (MSC) therapies appear to show promise in regenerative medicine [4]. MSCs are multipotent, non-haematopoietic stem cells, which can be collected from various sources (bone marrow (BM), liver, kidney, adipose tissue, urine, umbilical cord blood, umbilical tissue Wharton’s jelly, placenta) [27,28]. MSCs are identified by their expression of surface markers, CD73, CD90, and CD105, and lack of expression of haematopoietic markers (CD11b, CD19, CD34, CD45, CD79, HLA-DR) [29]. Owing to their multipotency, MSCs can replicate and differentiate into specialised cells to repopulate injured tissues [30,31]. However, emerging evidence has shown that they coordinate cellular communication and tissue repair by secreting bioproducts, called “extracellular vesicles” (EVs), which carry a diverse repertoire of trophic factors and genetic material [29,31,32]. The source of MSCs can affect their differentiation capacity and secretome [33]. For example, umbilical-MSCs share similar properties to BM-MSCs and retain primitive characteristics of embryonic stem cells [34]. Moreover, Wharton’s jelly MSCs are considered more immune-privileged and exert greater immunosuppressive properties compared to adipose tissue [35] or BM-MSCs [36]. Nonetheless, it is the ease of harvesting MSCs and their availability that determines their clinical applicability, and so both adult and fetal sources of MSCs will be discussed.

However, there are some disadvantages of using cell-based MSC therapy. These include the difficulty in generating a consistent source of cells with a stable phenotype and the issue of delivering large cells intravenously where there is a risk of entrapment in the pulmonary microvasculature, known as “first-past effect” [37]. Furthermore, there is a risk of MSCs inducing granulocytosis [38], graft rejection, ectopic tissue formation [39], and promotion of tumour growth [40,41]. By comparison, EVs can be prepared from the conditioned media of MSCs and offer greater safety, biological tolerance, easier tissue migration [42], lower propensity to induce an immune response [4], and no tumourigenicity [43].

## 4. Mesenchymal Stem Cell-Derived Extracellular Vesicles

**Figure 1:**
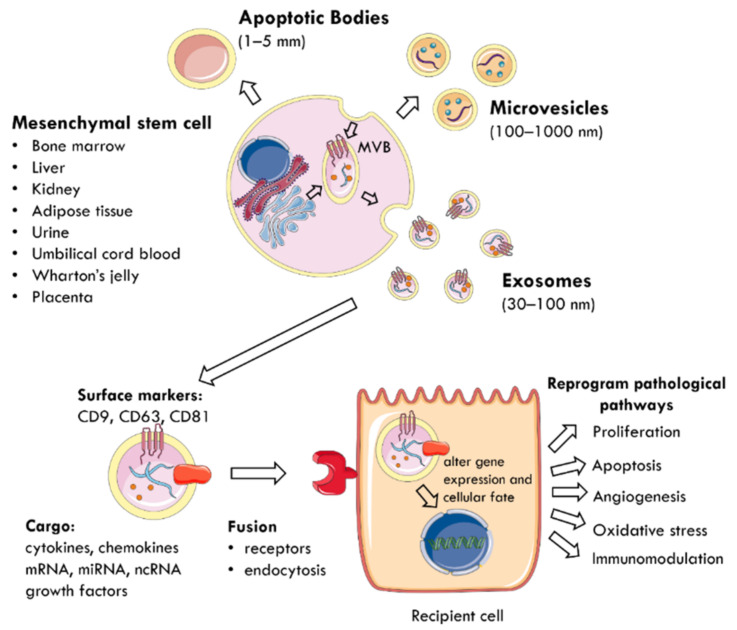
The mechanism by which EVs are secreted from MSCs and mediate paracrine communication. MSCs can be harvested from multiple tissue sources (bone marrow, liver, kidney, adipose tissue, urine, umbilical cord blood, Wharton’s jelly, placenta). EVs are membrane-enclosed particles classified into three categories: apoptotic bodies, microvesicles, and exosomes. For example, exosomes are secreted when MVB fuse with the plasma membrane and are characterised by surface expression of CD9, CD63, and CD81. They deliver a cargo of mRNA, miRNA, ncRNA, cytokines, chemokines, and growth factors to nearby injured cells. EVs utilise specific receptors or membrane fusion to enter target cells and the delivered material alters gene expression and cellular fate. This reprograms pathophysiological pathways, such as proliferation, apoptosis, angiogenesis, oxidative stress, and immunomodulation. MSC: mesenchymal stem cell; MVB: multivesicular bodies; EVs: extracellular vesicles; CD: cluster of differentiation; ncRNA: non-coding RNA.

The regenerative properties of MSCs are mediated by the paracrine action of EVs delivering biomolecules to neighbouring cells [40,43,44] (Figure 1). EVs are membrane-enclosed particles classified by their cellular origin into three categories: apoptotic bodies (1–5 mm), microvesicles (MVs) or microparticles (MPs) (100–1000 nm), and exosomes (30–100 nm) [4,45]. The biogenesis of these EV subtypes is distinct [43,46] (Figure 1). Exosomes are derivatives of the endosomal compartment, secreted into the environment when multivesicular bodies (MVB) fuse with the plasma membrane [44,47,48]. Given the difficulties in differentiating the subtypes of EVs by their size, the International Society for Extracellular Vesicles (ISEV) prefers the collective term of “EVs” to define particles released from cells that are bounded by a lipid bilayer and cannot replicate [49].

EVs carry a cargo reflecting the phenotype of their cells of origin [50], which includes cytokines, chemokines, growth factors, mRNA, miRNA, and other non-coding RNA [28,51,52]. The collection of surface proteins, originating from the endosomal pathway, distinguishes exosomes from MVs and includes tetraspanins (CD9, CD63, CD81), heat shock protein (HSP) 70, ALIX, and tumour suppressor gene 101 [27,40,49]. EVs use specific receptors or membrane fusion to enter target cells and deliver their contents as a form of paracrine communication [53]. Consequently, the delivered material modifies the phenotype of recipient cells by altering gene expression, stimulating transcription, inducing phenotypic switches, or determining cellular fate, self-renewal, and differentiation [29,52]. At the cellular level, this reprograms pathophysiological pathways, such as proliferation, apoptosis, oxidative stress, angiogenesis, and immunomodulation to promote tissue regeneration [4,45,50,54].

Therefore, this review will summarise the methodologies for isolating exosomes, the different clinical applications of MSC-EVs and, more specifically, their regenerative capacity in treating animal models of AKI and CKD.

## 5. Methodologies of Exosome Isolation

The therapeutic use of exosomes requires the development of methodologies that isolate exosomes of suitable quantity and quality from MSCs [43]. Exosomes can be collected from various biofluids, including plasma, serum, urine, and saliva [55] with conditioned cell culture media being the most widely used material [56]. Current isolation protocols are based on the physical properties of density and size, or chemically through the surface interactions with proteins [57] (Table 1). However, the methods are tedious and non-specific and there is no consensus on a gold standard of isolation [58], with differential ultracentrifugation chosen by 80% of researchers [56]. There is no set characterisation of the three populations of EVs by size, so most methods isolate a heterogeneous population [40,45,59,60]. Further standardisation is required.

## 6. Therapeutic Applications of MSC-EVs

In searching the database of https://clinicaltrials.gov (accessed on 18 May 2021), there are currently thirteen clinical trials investigating the therapeutic efficacy of MSC-EVs in various diseases. MSC-EVs have been applied in diabetes mellitus [40,70,71], myocardial infarction [43], conditioning transplants [72,73,74], cancer [42,75], macular degeneration [76,77], repairing bone defects [78], osteoarthritis [79], Alzheimer’s disease (AD) [42,80,81,82], ischaemic stroke [40,83,84], multiple sclerosis [85], and treating COVID-19 [86,87,88,89].

### 6.1. Macular Degeneration

An ongoing Phase I trial (NCT03437759) is investigating the healing capacity of umbilical-MSC-exosomes to repair areas of large and refractory macular injury [77]. A single dose of 20 μg or 50 μg exosomes will be applied around the injured macular area in 44 patients and the visual outcome will be followed up six months later. Pre-clinical studies propose that MSC-exosomes suppress inflammation and inhibit apoptosis by downregulating MCP-1, a key chemoattractant for monocytes [76].

### 6.2. Cancer

Tumour cells are known to secrete pathogenic exosomes to facilitate paracrine communication in the tumour microenvironment and promote tumour growth, invasion, metastasis, and drug resistance [90]. Many clinical trials have focused on the role of exosomes as diagnostic (NCT04394572) [91] or prognostic (NCT04288141) [92] indicators in cancer. However, MSC-exosomes may also be therapeutic in targeting cancers with known driver mutations. For example, MSC-exosomes engineered to carry siRNA specific to the oncogenic KrasG12D mutation (iExosomes) have successfully supressed pancreatic ductal adenocarcinoma in mice [93]. In a current Phase I trial (NCT03608631), these iExosomes will be delivered intravenously on days 1, 4, and 10 and repeated every 14 days for three courses of treatment for patients with Stage IV pancreatic ductal adenocarcinoma, harbouring the KrasG12D mutation [94].

### 6.3. Alzheimer’s Disease

The ability of MSC-EVs to cross the blood brain barrier means they could treat neurodegenerative diseases, such as AD [42]. EVs can directly internalise β-amyloid for lysosomal clearance [81], or transfer an insulin-degrading enzyme [95] or small interfering RNA [96] to reduce β-amyloid production, which is considered pathogenic in AD. A phase I/II trial (NCT04388982) is currently investigating the safety and efficacy of 5 μg, 10 μg, or 20 μg of allogenic adipose-MSC-exosomes administered to patients with AD twice a week for three months [82].

### 6.4. Ischaemic Stroke

As a result of shared risk factors, patients with Stage 4 CKD are twenty times more likely to die prematurely from cardiovascular events than progressing to end-stage kidney failure [9,20]. Preclinical studies in mice showed that miR-124-enriched exosomes induced neurogenesis, protected against ischaemic injury, and prevented post-ischaemic immunosuppression [84]. In an ongoing Phase II/III trial (NCT03384433), BM-MSC-exosomes, loaded with miR-124, will be administered to the ischaemic area one month following the stroke and the patient’s neurological outcome will be assessed one year later [83].

### 6.5. ARDS and COVID-19

The anti-inflammatory effect of MSC-exosomes is being investigated in coronavirus (SARS-CoV-2) pneumonia and acute respiratory distress syndrome (ARDS), where only supportive care exists [87]. A few clinical trials are currently investigating the efficacy of aerosol inhalation of allogenic adipose-MSC-exosomes in the treatment of ARDS over seven days (NCT04602104) [88] and intravenous delivery of an escalating dose of MSC-exosomes over five days in COVID-19 pneumonia (NCT04798716) [89]. The mechanism by which EVs elicit their immunomodulatory effects and whether they would be suitable therapeutic candidates is yet to be determined [97].

## 7. Nephroprotective Role of MSC-EVs in AKI

Multiple animal models have demonstrated that MSC-EVs can ameliorate AKI induced by cisplatin, glycerol, gentamicin, or ischaemic-reperfusion injury (IRI) [43,98,99]. Given the heterogeneous nature of MSC-EVs, it currently remains unknown which subtypes provide renoprotection, so the efficacy of MVs, MPs, and exosomes in rodent AKI will all be discussed (Table 2).

### 7.1. Tubular Proliferation and Dedifferentiation

Biodistribution analyses illustrate that MSC-EVs specifically accumulate at the site of injury and their cargo of growth factors determines regenerative capacity [100]. In glycerol- [101] or cisplatin-triggered [102] AKI and IRI [53,103,104], MSC-MVs delivered mRNA of the mesenchymal phenotype or IGF-1 receptor [102] to proximal TECs and this induced expression of hepatocyte growth factor (HGF) and macrophage-stimulating protein (MSP). This promoted proliferation and dedifferentiation of proximal TECs. Similarly, BM-MSC-EVs, enriched with pro-regenerative miRNA (miR-10a, miR-486), induced TEC proliferation, reduced BUN, creatinine, and proteinuria, and improved renal function following glycerol-triggered AKI [105]. Additionally, activation of the ERK1/2 pathway and downregulation of p38 MAPK signalling was attributed to increased cell proliferation and the reversal of cisplatin-mediated damage in kidneys treated with umbilical-MSC-exosomes [106] and BM-exosomes [107,108].

### 7.2. Inhibition of Apoptosis

Numerous studies show EVs protect tubular cells from apoptosis [32,98,101,104,109,110,111,112,113,114,115,116,117,118,119,120,121] and necrosis [98,105,106,109,122,123] following AKI. In severe combined immunodeficient (SCID) mice with cisplatin-induced AKI, a single injection of MSC-MVs increased survival to 40% at three weeks, compared to 100% mortality within five days of receiving the vehicle [109]. Multiple injections of MVs improved survival to 80% and restored renal function, indicating the dose response relationship. The mechanism of renoprotection was attributed to the upregulation of anti-apoptotic genes (Bcl-2, Bcl-xL, BIRC8) and downregulation of executioner genes (Caspase-1,-3,-8, lymphotoxin-α), thereby inhibiting apoptosis of proximal TECs [106]. Furthermore, BM-MSC-exosomes delivered miR-199a-3p into mice with IRI and suppressed apoptosis by downregulating Bax, Caspase-3, and the semaphorin, Sema3A [112]. Blocking Sema3A also led to activation of the Akt and ERK pathways for cell proliferation and offered protection against AKI. Similarly, BM-MSC-exosomes secreted miR-199a-5p, which targeted binding immunoglobulin protein (BIP) to inhibit endoplasmic reticulum stress in IRI within 8–16 h [123]. Another study demonstrated Wharton’s jelly MSC-EVs delivered miR-30b/c/d to injured TECs and mitigated DRP1-induced mitochondrial fragmentation caused by IRI and abrogated apoptosis [120].

### 7.3. Angiogenesis

The horizontal transfer of proangiogenic factors, such as vasculogenic growth factor (VEGF-A), IGF-1, and basic fibroblast growth factor (bFGF) from MSC-EVs to resident cells mediates nephroprotection by EVs [108,111,113,124]. The downregulation of HIF-1α led to increased density and perfusion of renal capillaries, thereby reducing hypoxia [111].

### 7.4. Anti-Oxidation

During ischaemia, ATP levels rapidly fall, whilst intracellular calcium, protons, and reactive oxygen species (ROS) levels and lactic acid rise [125,126]. Increased mitochondrial membrane permeability and release of lysosomal enzymes cause breakdown of TECs. BM-MSC-EVs reduced ischaemic damage in isolated rat kidneys by upregulating enzymes involved in cellular metabolism (Calbindin1) and ion membrane transport (Slc16a1, vacuolar H^+^-ATPase d2 subunit) [74]. Calbindin1 sequesters excess calcium and reduces ROS and apoptosis [127]. Slc16a1 encodes for monocarboxylate transporter 1 and exports accumulated lactic acid [128]. Additionally, H^+^-ATPase pumps protons across the cell membrane and this reduces intracellular acidosis [129]. Therefore, EV-treated kidneys had lower glucose but higher pyruvate levels compared to ischaemic kidneys [74], indicating the important anti-oxidant activity of EVs [126].

The anti-oxidant activity of EVs may involve upregulation of nuclear factor E2-related factor (Nrf2) [130], which is a transcription factor binding anti-oxidant response elements and improves the expression of ROS scavenging enzymes, such as superoxide dismutase (SOD) and heme oxygenase-1 (HO-1) [131]. Wharton’s jelly MSC-EVs [130] and -MVs [119] alleviated oxidative stress in rats with unilateral kidney ischaemia and IRI, respectively, by downregulating NOX2 expression, which is a NADPH oxidase generating ROS. The authors hypothesised that miRNA delivered by EVs activated Nrf2 [130] or suppressed NOX2 expression [119]. Another IRI study supports this mechanism where human placenta-MSC-EVs delivered a cargo of miR-200a-3p to TECs [132]. The miRNA downregulated Keap1, freeing Nrf2 for nuclear translocation and promoted SOD2 expression. This reinforced antioxidant defence, increased ATP production, and protected TECs from mitochondrial fragmentation.

Melatonin is a strong scavenger of ROS and exosomes derived from MSCs conditioned with melatonin reduced oxidative stress within three days of IRI [133]. Exosomes carried RNA that downregulated expression of ROS such as malondialdehyde, HIF-1α, and NOX2, and upregulated anti-oxidant molecules (HO-1, SOD, catalase, glutathione peroxidase) [113].

### 7.5. Immunomodulation

The anti-inflammatory effects of EVs can be attributed to their paracrine delivery of immunomodulatory molecules or expression of surface proteins that minimises infiltration of immune cells, such as macrophages, T cells, and NK cells [134]. BM-MSC-exosomes downregulated pro-inflammatory cytokines (IL-6, IL-1β, IFN-γ, TNF-α) and stimulated anti-inflammatory cytokines (IL-10) in rodents with IRI [113], unilateral ureteral obstruction (UUO) [135], or gentamicin- [98] or cisplatin-induced AKI [107,108,136]. Additionally, umbilical-MSC-exosomes upregulated miR-146b, leading to reduced IRAK1 expression and NF-κB transcriptional activity in TECs in mice with sepsis-associated AKI [137]. By dampening the cytokine storm of sepsis, exosomes alleviated AKI and improved survival from 28% to 45% at day three. Recent studies have demonstrated the synergistic role of pulsed focused ultrasound and BM-MSC-EVs in reducing HSP70 expression and inhibiting activation of the NRLP3 inflammasome and its pro-inflammatory cytokines (IL-1β, IL-18) in cisplatin-induced AKI [108,136].

As mentioned earlier, Wharton’s jelly MSCs exert greater immunomodulatory activity than other sources of MSCs [35,36]. A single injection of Wharton’s jelly MSC-MVs delivered miR-15a/-15b/-16 to rats with IRI and this suppressed CX3CL1 expression and CD68+ macrophage infiltration [110,118]. Furthermore, surface expression of CCR2 by exosomes could sequester its extracellular ligand, CCL2, and interfere with macrophage recruitment in IRI [134]. Additional studies demonstrated Wharton’s jelly MSC-EVs decrease NK cell infiltration in ischaemic kidneys through downregulation of CX3CL1 and TLR2 expression [138]. This immunomodulation was preserved in rats with a splenectomy, indicating the spleen was not necessary for EVs to mediate renoprotection, unlike MSCs.

In summary, MSC-EVs ameliorate AKI by inducing tubuloepithelial regeneration and angiogenesis, and dampening apoptosis, oxidative stress, and inflammation [4,139].

**Table 2:** Comparison of EVs subtypes from various sources of MSCs in treating rodent models of AKI. Molecules critical for MSC-EVs to mediate nephroprotection are emboldened. Up (↑) indicates increased, and down (↓) indicates decreased levels or activity.

MSC Source	In Vivo Model	EV Subtype	Dose	Administration	Pathophysiological Effects	Mechanism of Action	Ref.
Bone marrow	Glycerol	EVs	Single: 200 μg	Intravenous	EVs accumulate specifically in injured kidneys		[100]
Bone marrow	Glycerol	MVs	Single: 15 μg	Caudal vein	MVs accumulated within lumen of injured tubules ↑ proliferation ↓ apoptosis ↑ tubuloepithelial regeneration	Delivery of **HGF**, **MSP**	[101]
Bone marrow	Glycerol	EVs	Single: 16.5 × 10^7^ or 8.25 × 10^7^	Intravenous	Pro-regenerative miRNA-enriched EVs are superior to naïve EVs at lower doses ↓ BUN, creatinine ↓ necrosis	Pro-regenerative miRNA: **miR-10a**, **miR-486**, **miR-127**	[105]
Bone marrow	Cisplatin	MVs	Multiple: 100 μg, then 50 μg days 2, 6, 10, 14, 18	Intravenous	↓ apoptosis, necrosis ↑ proliferation ↓ mortality Did not prevent chronic tubular injury at 3 weeks	↓ Caspase-1,8, lymphotoxin-α ↑ Bcl-2, Bcl-xL, BIRC8	[109]
Bone marrow	Cisplatin	EVs	Single: 150 μg	Intra-arterial kidney	↓ BUN, creatinine ↓ tubular cast formation ↑ proliferation ↓ inflammation	↓ IL-6, TNF-α, NF-κB	[107]
Bone marrow	Cisplatin	EVs	Single: 200 μg/100 g body weight on day 3	Intraperitoneal	Combined pre-treatment with pulsed focused ultrasound on d2 ↓ BUN, creatinine ↓ tissue damage (KIM-1, NGAL) ↓ inflammation	↓ HSP70, HSP90 activation of **NLRP3** inflammasome ↓ IL-1β, IL-18	[136]
Bone marrow	Cisplatin	EVs	Single: 150 μg/100 g body weight on day 3	Caudal vein	Pulsed focused ultrasound pre-treatment ↓ tissue damage (KIM-1, TIMP-1) ↑ proliferation ↑ angiogenesis ↓ apoptosis ↓ inflammation	↑ **ERK** signalling ↑ **PI3K/Akt** ↑ VEGF, PCNA, survivin ↑ **SIRT3**, **eNOS** ↓ Caspase-3, Bax ↓ TNF-α, IL-6, IL-1β	[108]
Bone marrow	Gentamicin	Exosomes	Multiple: 100 μg	Caudal vein	↓ apoptosis, necrosis ↑ proliferation ↓ inflammation	Unknown RNA ↓ IL-6, IFN-γ, TNF-α; ↑ IL-10	[98]
Bone marrow	IRI	Exosomes	Single: 200 μg	Renal capsule	↓ macrophage infiltration ↓ inflammation	**CCR2** expression on exosomes suppress CCL2 activity	[134]
Bone marrow	IRI	Exosomes preconditioned with 5 μM **melatonin**	Single: 250 μg	Perfusion	↓ BUN, creatinine ↓ apoptosis ↓ oxidative stress ↓ inflammation ↑ regeneration ↑ angiogenesis	**Melatonin:** ↓ Caspase-3, Bax, PARP1; ↑ Bcl-2 ↓ ROS: MDA, HIF-1α, NOX2 ↑ anti-oxidants (HO-1, SOD, CAT, GPX) ↓ MPO activity, ICAM-1, IL-1β, NF-κB; ↑ IL-10 ↑ bFGF, HGF, Sox9, VEGF	[113]
Bone marrow	IRI	Exosomes enriched with **miR-199a-3p**	Single: 5 × 10^5^	Caudal vein	↓ apoptosis	↓ Sema3A and reactivate **Akt** and **ERK** pathways ↓ Caspase-3	[112]
Bone marrow	IRI	Exosomes enriched with **miR-199a-5p**	Single: 5 × 10^5^	Caudal vein	↓ endoplasmic reticulum stress at 8–16 h after reperfusion ↓ apoptosis	Targets **BIP**	[123]
Bone marrow	IRI, nephrectomy	EVs	Single: released from 3 × 10^6^ MSCs	Perfusion	↓ ischaemic damage	↑ Expression of proteins in membrane transport and homeostasis (Calb1, Slc16a1, vaculor H^+^-ATPase d2 subunit)	[74]
Bone marrow	UUO	EVs	Single: 0.5 mg/kg	Intravenous	↓ inflammation ↓ macrophage infiltration (ED-1+) ↓ mitochondrial damage ↓ oxidative stress ↓ apoptosis ↓ fibrosis	Delivered **MFG-E8** to inhibit **RhoA/ROCK** pathway ↓ IL-1β, TNF-α, IL-6 ↓ MDA; ↑ anti-oxidants (SOD, CAT) ↓ Caspase-3, PARP1 ↓ α-SMA, ↓fibronectin, ↑E-cadherin	[135]
Umbilical cord	Cisplatin	Exosomes	Single: 200 μg	Renal capsule	↓ apoptosis, necrosis ↓ oxidative stress ↑ proliferation	↓ Caspase 3 ↓ **p38 MAPK** pathway	[106]
Umbilical cord	Cisplatin	Exosomes	Single: 200 μg	Renal capsule	↑ autophagy: ↑LC3B ↓ BUN, creatinine after 3d ↓ apoptosis ↓ inflammation	↓ **mTOR** activity ↓ Bax, ↓ Caspase-3; ↑ Bcl-2, Bcl-XL ↓ IL-1β, IL-6, TNF-α	[114]
Umbilical cord	Cisplatin	Exosomes	Single: 200 μg	Renal capsule	↑ autophagy ↓ BUN, creatinine after 3d ↓ apoptosis	Delivered **14–3-3ζ** to ↑ autophagy via promoting the localisation of ATG16L ↓ Caspase 3	[115]
Umbilical cord	IRI	EVs overexpressing **Oct4**	Single: 100 μg	Caudal vein	↓ BUN, creatinine ↓ apoptosis ↑ proliferation ↓ fibrosis	**Oct4** inhibited fibrosis (↓ SNAIl, α-SMA)	[117]
Umbilical cord	Sepsis (caecal ligation and puncture)	Exosomes	Single: 120 μg	Caudal vein	↓ BUN, creatinine ↓ apoptosis ↓ inflammation ↑ survival (45% vs. 28% control)	Upregulation of **miR-146b** ↓ IRAK1 and ↓ NF-κB expression ↓ IL-1β, TNF-α	[137]
Wharton’s jelly	IRI	MVs	Single: 100 μg	Caudal vein	↓ BUN, creatinine ↓ apoptosis ↑ tubular cell proliferation ↓ inflammation ↓ CD68^+^ macrophage infiltration ↓ fibrosis	Delivery of miRN-**15a/-15b/-16** reduced CX3CL ↓ α-SMA	[110]
Wharton’s jelly	IRI	MVs	Single: 100 μg	Caudal vein	↓ oxidative stress ↓ apoptosis ↑ proliferation ↓ fibrosis	↓ **NOX2** expression, ↓ ROS levels ↓ α-SMA	[119]
Wharton’s jelly	IRI	MVs	Single: 30 μg	Caudal vein	↑ tubular cell dedifferentiation and growth	↑ **HGF** RNA	[103]
Wharton’s jelly	IRI	MVs	Single: 100 μg	Intravenous	↑ survival ↓ BUN, creatinine ↓ apoptosis ↑ proliferation ↓ inflammation ↓ CD68+ macrophage infiltration ↓ fibrosis	↓ TNF-α; ↑ IL-10 ↓ α-SMA, TGF-β1 ↑ HGF	[118]
Wharton’s jelly	IRI	EVs	Single: 100 μg	Intravenous	↓ BUN, creatinine after 24 h ↓ NK cells in kidney without the involvement of the spleen	↓ CX3CL1, TLR2	[138]
Wharton’s jelly	IRI	EVs	Single: 100 μg	Caudal vein	↑ angiogenesis ↓ fibrosis	Delivery of **VEGF** and its RNA; ↓ HIF-1α, α-SMA	[124]
Wharton’s jelly	IRI	EVs	Single: 100 μg	Caudal vein	↓ mitochondrial fission ↓ apoptosis	Delivery of **miR-30b/c/d**	[120]
Wharton’s jelly	IRI	EVs	Single: 100 μg	Caudal vein	↓ oxidative stress↓ renal cell injury (↓NGAL) ↓ apoptosis	↑ **Nrf2/ARE** activation ↑ ROS scavenging enzymes (HO-1)	[130]
Renal	IRI	EVs	Single: 4 × 10^8^	Intravenous	EVs detected in ischaemic kidneys within 1 h ↓ BUN, creatinine ↑tubular cell proliferation	Identified 62 **miRNAs**	[53]
Renal	IRI	EVs	Single: 2 × 10^7^	Caudal vein	↓ apoptosis ↑ peritubular capillary endothelial cell proliferation ↑ angiogenesis	Selective engraftment in ischaemic kidneys Delivery of **VEGF-A**, **bFGF**, **IGF-1**	[111]
Adipose	IRI	Exosomes	Single: 100 μg	Intravenous	Combined ADMSC and exosome therapy is superior to monotherapy: ↓ proteinuria ↓ kidney injury score		[140]
Adipose	Sepsis (caecal ligation and puncture)	Exosomes	Single: 100 μg	Caudal vein	↓ inflammation ↓ inflammatory cell infiltration ↓ apoptosis ↓ mortality	↑ **SIRT1** inhibited NF-κB and its inflammatory activity ↓ TNF-α, IL-6, MCP-1 ↓ Bax, ↓ Caspase-3; ↑ Bcl-2	[141]
Human induced pluripotent stem cells	IRI	EVs	Single: 1 × 10^12^	Intravenous	↓ necroptosis	Delivery of **SP1** to renal cells	[122]
Human placenta-derived	IRI	EVs	Single: 80 μg	Intravenous	EVs specifically accumulated in ischaemic kidney and taken up by proximal TECs ↑ mitochondrial antioxidant defence ↓ mitochondrial fragmentation	**Keap1-Nrf2** pathway- ↑ SOD2, ↑ATP production	[132]
Human placenta-derived	IRI	EVs	Multiple: 100 μg daily for 3 days		EVs travelled to injured kidneys ↑ proliferation and regeneration ↓ BUN, creatinine ↓ apoptosis ↓ fibrosis d28	↑ **Sox9**+ expression in tubular epithelial cells ↓ α-SMA, fibronectin, collagen I, TGF-β1	[142]

Abbreviations: MSC: mesenchymal stem cell; AKI: acute kidney injury; EVs: extracellular vesicles; MVs: microvesicles; miR: miRNA; HGF: hepatocyte growth factor; MSP: macrophage-stimulating protein; BUN: blood urea nitrogen; Bcl-2: B-cell lymphoma 2; Bcl-xL: B-cell lymphoma-extra-large; BIRC8: baculoviral IAP repeat containing 8; IL-6: interleukin-6; TNF-α: tumour necrosis factor alpha; NF-κB: nuclear factor kappa-light-chain enhancer of activated B cells; KIM-1: kidney injury molecule-1; NGAL: neutrophil gelatinase-associated lipocalin; HSP: heat shock protein; NRLP3: NLR family pyrin domain containing 3; ERK: extracellular signal-regulated kinase; PI3K: phosphoinositide 3-kinase; Akt: protein kinase B; TIMP-1: tissue inhibitor matrix metalloproteinase 1; VEGF: vascular endothelial growth factor; PCNA: proliferating cell nuclear antigen; SIRT3: sirtuin 3; p-eNOS: phosphorylated endothelial nitric oxide synthase; IRI: ischaemia-reperfusion injury; IFN-γ: interferon-γ; CCR2: C-C motif chemokine receptor type 2; CCL2: C-C motif chemokine ligand 2; PARP1: poly [ADP-ribose] polymerase 1; ROS: reactive oxygen species; MDA: malondialdehyde; HIF-1α: hypoxia-inducible factor 1 alpha; NAPDH: nicotinamide-adenine dinucleotide phosphate; NOX2: NADPH oxidase 2; HO-1: haeme oxygenase 1; SOD: superoxide dismutase; CAT: catalase; GPX: glutathione peroxidase; MPO: myeloperoxidase; ICAM-1: intercellular adhesion molecule 1; bFGF: basic fibroblast growth factor; Sox9: SRY-box transcription factor 9; Sema3A: semaphorin-3A; BIP: binding immunoglobulin protein; Calb1: calbindin 1; Slc16a1: solute carrier family 16 member 1; UUO: unilateral ureteral obstruction; MFG-E8: milk fat globule-EGF factor 8 protein; RhoA: Ras homolog family member A; ROCK: Rho-associated protein kinase; α-SMA: alpha smooth muscle actin; MAPK: mitogen-activated protein kinase; LC3: microtubule-associated protein light chain 3; mTOR: mammalian target of rapamycin; ATG16L: autophagy related 16 like 1; CX3CL: C-X3-C motif chemokine ligand 1; TLR: toll-like receptor; Oct4: octamer-binding transcription factor 4; SNAI1: snail family transcriptional repressor 1; IRAK1: interleukin-1 receptor associated kinase 1; CD68: cluster of differentiation; TGF-β: transforming growth factor beta; ROS: reactive oxygen species; Nrf2: nuclear factor erythroid 2-related factor 2; ARE: antioxidant response element; IGF-1: insulin growth factor-1; ADMSC: adipose-derived mesenchymal stem cell; MCP-1: monocyte chemoattractant protein-1; SP1: proximal specificity protein 1; TEC: tubular epithelial cells; ATP: adenosine triphosphate; Keap1: Kelch-like ECH-associated protein 1.

## 8. Anti-Fibrotic Effect of MSC-EVs in CKD

Renal glomerulosclerosis and tubulointerstitial fibrosis are hallmarks of diabetic nephropathy and indeed all types of CKD [4]. MSC-EVs promote tissue regeneration by targeting kidney fibrosis, reducing tubular atrophy and inflammation, and facilitating angiogenesis to abrogate pathogenic insults in CKD (Table 3).

### 8.1. Downregulate Pro-Fibrotic Gene Expression and the EMT

TGF-β is a key inducer of the EMT in CKD [12]. In β-integrin signalling, TGF-β forms a complex with Smad and binds transcription factors, such as Snail, to downregulate expression of epithelial cell markers (E-cadherin), and activate expression of fibrosis-associated stromal cell markers (α-SMA, fibronectin, collagen I) [143]. Erythropoietin (EPO)-transfected microparticles in mice with UUO [144,145], Wharton jelly’s MSC-EVs in cyclosporin A injury [146], and adipose-MSC-EVs in renal artery stenosis [147] all inhibited the EMT and tubulointerstitial fibrosis by downregulating phosphorylated Smad2, Smad3, and p38 MAPK signalling, and increasing E-cadherin.

The anti-fibrotic mechanism of MSC-EVs is mediated through the transfer of miRNA targeting fibrosis-associated genes [4,54,116,117,148]. For example, MSC-exosomes delivered miRNA-let7c to mouse TECs with UUO and downregulated the expression of collagen IVα1, metalloproteinase-9 (MMP9), α-SMA, and TGF-β1 and its receptor [149]. Similarly, NOD SCID gamma mice with streptozotocin (STZ)-induced T1DM were treated with BM-MSC-EVs and their cargo of miRNA suppressed fibrotic (collagen I, MMP3, TIMP1) and apoptotic (FasL, Serpina1a) gene expression [54]. Umbilical-MSCs-MVs, enriched with miR-451a, reversed the EMT in STZ-induced diabetic nephropathy by increasing E-cadherin expression and reducing fibrosis [150]. The shuttled miR-451a targeted the 3′UTR sites of cell cycle inhibitors, P15INK4b and P19INK4d, enabling a resumption of the blocked cell cycle and amelioration of the EMT. In aristolochic acid-induced herbal nephropathy, BM-MSC-EVs reduced tubular necrosis and interstitial fibrosis by suppressing expression of fibrotic genes (α-SMA, TGF-β1, collagen Iα1) [151]. This was postulated by MSC-EVs downregulating various miRNAs (miR21-5p, 34a-5p, 34c-5p, 132-3p, 212-3p, 214-3p, 342-3p) that mediate fibrosis, inflammation, and apoptosis. Hence, EVs can revert the progression of tubulointerstitial fibrosis and the EMT and restore function in CKD [16,54,152].

### 8.2. Reduce Tubular Atrophy

EVs exhibit anti-apoptotic activity to prevent the transition of AKI into CKD [109,145,153]. Six months following treatment with umbilical-MSC-MVs, rats with IRI showed dwindling tubular atrophy, improved functioning, and decreased glomerular ECM accumulation and fibrosis [104]. Reduced-to-absent tubular atrophy and repaired renal morphology were also observed in mice with 5/6 subtotal nephrectomy treated with MSC-MVs [153]. Furthermore, BM-MSC-EVs reduced degeneration, vacuolisation, tubular cyst formation, and atrophic changes of proximal TECs in mice with T1DM, T2DM [16], or cyclosporin nephrotoxicity [154].

### 8.3. Vascular Regeneration

Urinary MSC-exosomes delivered VEGF, TGF-β1, angiogenin, and BMP7 for vascular and tubular regeneration in STZ-induced T1DM [121]. Renal MSC-microparticles offered similar protection by attenuating TGF-β1-induced endothelial-to-mesenchymal transition of PTC, thereby improving capillary density and reducing fibrosis in kidneys with UUO [155].

A high cholesterol/fructose diet induces Metabolic Syndrome that not only increases the risk of CKD progressing to end-stage kidney failure [156], but also hinders the proliferative and differentiation potential of MSCs [157]. In a swine model of unilateral renovascular disease and Metabolic Syndrome, autologous adipose-MSC-EVs, enriched with pro-angiogenic factors (VEGF-A,C, VEGF receptor, angiopoietin-like 4, HGF), were internalised by tubular and endothelial cells within four weeks and improved cortical microvascular density, renal blood flow, and GFR [158].

### 8.4. Anti-Inflammatory

Intercellular adhesion molecule 1 (ICAM-1) is a glycoprotein expressed by TECs and PTCs to support the recruitment of inflammatory cells into injured kidneys [159]. In T1DM and T2DM mice, BM-MSC-exosomes reduced expression of ICAM-1 in PTCs and reversed infiltration of dendritic cells, thereby preventing the development of diabetic nephropathy [16]. Additionally, BM-MSC-EVs downregulated CCL3 and hindered recruitment of macrophages and T cells [54,160]. Another study demonstrated that adipose-MSC-EVs delivered miR-26a-5p to inhibit TLR4 and the NF-κB/VEGFA inflammatory pathway, thereby alleviating diabetic nephropathy [161]. BM-MSC-EVs reduced TNF-α expression and inflammation, leading to an improvement in CKD outcomes [16].

TGF-β induces gene expression of forkhead box-P3 (FoxP3) to create a population of regulatory CD4+ T cells (Tregs) that police excessive inflammation and this can be utilised to ameliorate CKD [162]. For example, MSC-EVs, harvested from lean pigs, upregulated TGF-β expression and induced Treg differentiation in pigs with Metabolic Syndrome and unilateral renal artery stenosis, thereby decreasing inflammation and tubulointerstitial fibrosis [163]. Lean-EVs shifted the balance of macrophages from a pro-inflammatory M1 to anti-inflammatory M2 phenotype, and reduced the numbers of cytotoxic CD8+ T cells and IL-1β expression. By contrast, MSC-EVs, derived from pigs with Metabolic Syndrome, failed to alleviate CKD. This indicates the importance of the source and phenotype of MSC-EVs, where Metabolic Syndrome altered the cargo of 19 mitochondria-related miRNAs and therefore impaired the therapeutic efficacy of EVs [164].

Ultimately, MSC-EVs offer nephroprotection against CKD through reversing fibrosis, reducing the EMT, inhibiting apoptosis, promoting angiogenesis, and suppressing inflammation.

**Table 3:** Comparison of EVs subtypes from various sources of MSCs in treating rodent models of CKD. Molecules critical for MSC-EVs to mediate nephroprotection are emboldened. Up arrow (↑) indicates increased and down arrow (↓) indicates decreased levels or activity.

MSC Source	In Vivo Model	EV Subtypes	Dose	Administration	Pathophysiological Effects	Mechanism of Action	Ref.
Bone marrow	IRI	MVs	Single: 30 μg	Intravenous	↓ BUN, creatinine, proteinuria ↓ fibrosis, ↓glomerular matrix accumulation ↓ interstitial lymphocyte infiltrate ↓ tubular atrophy	Dependent on RNA cargo	[104]
Bone marrow	Chronic CsA	EVs	Multiple: 100 μg Preventive: 24 h after CsA, weekly for 4 weeks Curative: 2 weeks after CsA, weekly for 4 weeks	Intraperitoneal	Greater improvement when administered after damage (curative regime), rather than prophylactically ↓ tubular casts	↓ PAI-1, TIMP-1, IFN-γ	[154]
Bone marrow	Aristolochic acid	EVs	Single: 1 × 10^10^ on day 3	Intravenous	↓ BUN, creatinine ↓ necrosis ↓ CD45+ immune cells, fibroblast, pericyte infiltration ↓ interstitial fibrosis	Downregulation of **hsa-miR-21-5p**, **34a-5p**, **34c-5p**, **132-3p**, **214-3p**, **342-3p**; and **mmu-miR-212-3p** Upregulation of **hsa-miR-194-5p**, **192-5p**; and **mmu-miR-378-3p** ↓ α-SMA, TGF-β1, collagen Iα1	[151]
Bone marrow	5/6 subtotal nephrectomy	MVs	Multiple: 30 μg, days 2, 3, 5	Caudal vein	↓ BUN, creatinine, uric acid, proteinuria prevent fibrosis ↓ tubular atrophy ↓ interstitial lymphocyte infiltrate		[153]
Bone marrow	UUO	MVs	Single: 30 μg	Caudal vein	↓ BUN, creatinine ↓ fibrosis	↓ TGF-β1, α-SMA ↑ E-cadherin	[152]
Bone marrow	UUO	Exosomes enriched with **miR-let7c**	Single: released from 1 × 10^6^ MSCs	Intravenous	Exosomes home to injured kidneys ↓ fibrosis	Delivery of **miRNA-let7c** ↓ collagen, MMP-9, α-SMA, TGF-βR1	[149]
Bone marrow	Type 2 diabetes, STZ Type 1 diabetes	Exosomes	Single: 5.3 × 10^7^	Renal subcapsular	↓ degeneration, vacuolation and tubular atrophy ↓ EMT ↓ ICAM-1-mediated interstitial inflammatory infiltration	↓ TGF-β ↓ TNF-α	[16]
Bone marrow	STZ Type 1 diabetes	Exosomes	Single: 100 μg	Intravenous	↑ Autophagy: ↑ LC3-II, Beclin-1 ↓ BUN, creatinine, blood glucose, proteinuria at 10 and 12 weeks ↓ fibrosis	↓ **mTOR** activity ↓ collagen, TGF-β	[165]
Bone marrow, Liver	STZ Type 1 diabetes	EVs	Multiple: 1 × 10^10^	Intravenous	↓ BUN, creatinine ↓ fibrosis, ↓ EMT ↓ inflammatory cell recruitment	↓ collagen I, MMP3, TIMP1, FasL, Serpina1a, SNAI1 ↓ CCL3	[54]
Umbilical cord	STZ-induced DN with hyperuricaemia	MVs enriched with **miR-451a**	Single: 1.5 mg/kg	Caudal vein	↓ BUN, creatinine ↓ fibrosis, ↓ EMT ↑ proliferation and removed arrest on cell cycle	↓ α-SMA, ↑ E-cadherin **miR-451a** targeted 3′UTR sites of cell cycle inhibitors (P15INK4b, P19INK4d)	[150]
Umbilical cord	UUO	Exosomes	Single: 200 μg	Intravenous	↓ tubulointerstitial fibrosis	Exosomes delivered casein kinase 1δ and E3 ubiquitin ligase β-TRCP to degrade **YAP**	[166]
Umbilical cord	UUO	Exosomes	Single: 200 μg	Intra-arterial kidney	↓ BUN, creatinine ↓ apoptosis ↓ oxidative stress ↓ tubulointerstitial fibrosis	↓ ROS-mediated **p38 MAPK/ERK** signalling pathway ↓ ROS: MDA ↑ anti-oxidants: GSH	[145]
Wharton’s jelly	CsA	EVs	Multiple: 100 μg at day 7, 21	Intravenous	↓ creatinine ↓ fibrosis, ↓ EMT ↓ oxidative stress	↓ α-SMA ↓ ROS: MDA ↑ anti-oxidants: SOD	[146]
Renal	UUO	MPs	Single: 2 × 10^7^	Caudal vein	↓ EndoMT of PTC endothelial cells ↓ PTC rarefaction ↓ F4/80+ inflammatory cell infiltration ↓ tubulointerstitial fibrosis	↓ α-SMA	[155]
Renal	UUO	EPO-enriched MPs	Single: 80 μg	Caudal vein	↓ tubulointerstitial fibrosis, ↓ EMT ↓ myofibroblast and F4/80+ macrophage infiltration	↓ phosphorylated **Smad2**, **Smad3**, **MAPK 38** expression to inhibit EMT ↓ α-SMA, fibronectin, collagen	[144]
Adipose (transfected with **GDNF**)	UUO	Exosomes	Single: 200 μg	Caudal vein	↓ PTC rarefaction ↓ tubulointerstitial fibrosis ↑ endothelial function and angiogenesis	**GDNF:** ↑ **SIRT1/p-eNOS** pathway ↓ α-SMA ↑ VEGF, ↓ HIF-1α	[167]
Adipose	IRI	Exosomes	Single: 100 μg	Caudal vein	↑ tubular proliferation, regeneration ↓ TGF-β1-induced transformation of TECs to pro-fibrotic phenotype ↓ AKI to CKD transition	↑ **Sox9** ↓ α-SMA, PDGFR-β	[168]
Adipose	Type 1 diabetes	Exosomes	Single: not stated, 12-week therapy	Caudal vein	↓ BUN, creatinine, proteinuria ↑ autophagy, ↓ apoptosis podocytes	**miR-486** reduced Smad1 expression, leading to ↓ **mTOR** activation	[169]
Adipose	Hindlimb Ischaemia	**Melatonin**-stimulated exosomes	CKD-MSCs treated with 30 μg exosomes, and 1 × 10^6^ cells injected	Injection into ischaemic site	CKD-MSCs were treated with **melatonin**-stimulated exosomes and injected into mice ↑ neovascularisation ↑ functional recovery	Upregulation of **miR-4516** ↑ PrP^c^ in exosomes	[170]
Adipose	DN (C57BL/KsJ db/db)	EVs	Single	Caudal vein	↓ histopathology of DN, ↓ BUN, creatinine ↓ VEGFA leads to ↓ podocyte apoptosis	**miR-26a-5p** inhibited TLR4 and inactivated NF-κB/VEGFA pathway (↓ IKKβ, IκBα, p65) ↓ Caspase-3, Bax, ↑ Bcl-2	[161]
Adipose	Unilateral renovascular disease on background of Metabolic Syndrome	EVs	Single: 1 × 10^7^	Intra-renal vein	↑ cortical microvascular, PTC density ↑ RBF, GFR ↓ glomerular, tubulointerstitial fibrosis ↓ apoptosis ↓ oxidative stress	Delivered proangiogenic factors: **VEGF-A,C, VEGF receptor**, **angiopoietin like 4, HGF** ↓ Caspase-3 ↓ ROS: superoxides, CD31, nitro tyrosine	[158]
Adipose	Unilateral renal artery stenosis on background of Metabolic Syndrome	EVs	Single: 1 × 10^10^	Intrarenal artery	EVs derived from lean pigs were injected into pigs with Metabolic Syndrome	↑ TGF-β induction of Tregs ↓ IL-1β	[163]
					↑ anti-inflammatory M2 macrophages		
					↓ pro-inflammatory M1 macrophages		
					↓ CD8+ T cells		
Adipose	Unilateral renal artery stenosis on background of Metabolic Syndrome	EVs	Single: 1 × 10^10^	Intrarenal artery	Metabolic Syndrome alters the cargo of 19 mitochondria-related miRNA, impairing regenerative capacity	↑ **miR-196a**, **132** ↓ **miR-192**, **320**	[164]
Adipose	Unilateral renal artery stenosis	MVs, exosomes	Single: 100 μg	Caudal vein	↓ HIF-1α Stabilised systolic blood pressure ↓ proteinuria (MVs only) ↑ natriuresis (exosomes only) ↓ fibrosis ↓ inflammation	↓ collagen I, TGF-β ↑ IL-10	[147]
Urine	STZ Type 1 diabetes	Exosomes	Multiple: 100 μg weekly x × 12	Intravenous	↓ apoptosis of podocyte and tubular cells ↑ glomerular endothelial cell proliferation ↑ angiogenesis	↓ Caspase-3 Delivery of **VEGF, TGF-β1, angiogenin, BMP7**	[121]

Abbreviations: MSC: mesenchymal stem cell; CKD: chronic kidney disease; EVs: extracellular vesicles; IRI: ischaemia-reperfusion injury; MVs: microvesicles; BUN: blood urea nitrogen; CsA: cyclosporin A; PAI-1: plasminogen activator inhibitor-1; TIMP-1: tissue inhibitor matrix metalloproteinase 1; IFN-γ: interferon-γ; α-SMA: alpha smooth muscle actin; TGF-β: transforming growth factor beta; miR: miRNA; CD45: cluster of differentiation 45; UUO: unilateral ureteral obstruction; MMP: matrix metalloproteinase; STZ: streptozotocin; ICAM-1: intercellular adhesion molecule 1; TNF-α: tumour necrosis factor alpha; EMT: epithelial-to-mesenchymal transition; LC3-II: microtubule-associated protein light chain 3; mTOR: mammalian target of rapamycin; Sepina1a: serpin family A member 1; SNAI1: snail family transcriptional repressor 1; CCL3: C-C motif chemokine ligand 3; DN: diabetic nephropathy; β-TRCP: β-transducin repeats-containing protein; YAP: yes-associated protein; MAPK: mitogen-activated protein kinase; ROS: reactive oxygen species; MDA: malondialdehyde; GSH: glutathione; SOD: superoxide dismutase; MPs: microparticles; EndoMT: endothelial-to-mesenchymal transition; PTC: peritubular capillaries; EPO: erythropoietin; GDNF: glial cell line-derived neurotrophic factor; VEGF: vascular endothelial growth factor; Bcl-2: B-cell lymphoma 2; Bax: Bcl-2-associated X protein; HIF-1α: hypoxia-inducible factor 1 alpha; Sox9: SRY-box transcription factor 9; TEC: tubular epithelial cells; PDGFR: platelet-derived growth factor receptor; AKI: acute kidney injury; PrP^c^: cellular prion protein; TLR: toll-like receptor; NF-κB: nuclear factor kappa-light-chain enhancer of activated B cells; HGF: hepatocyte growth factor; RBF: renal blood flow; GFR: glomerular filtration rate; Tregs: regulatory T cells; IL-1β: interleukin-1β; BMP7: bone morphogenetic protein 7.

## 9. Biological Cargo Carried by MSC-EVs to Alleviate AKI and CKD

**Figure 2:**
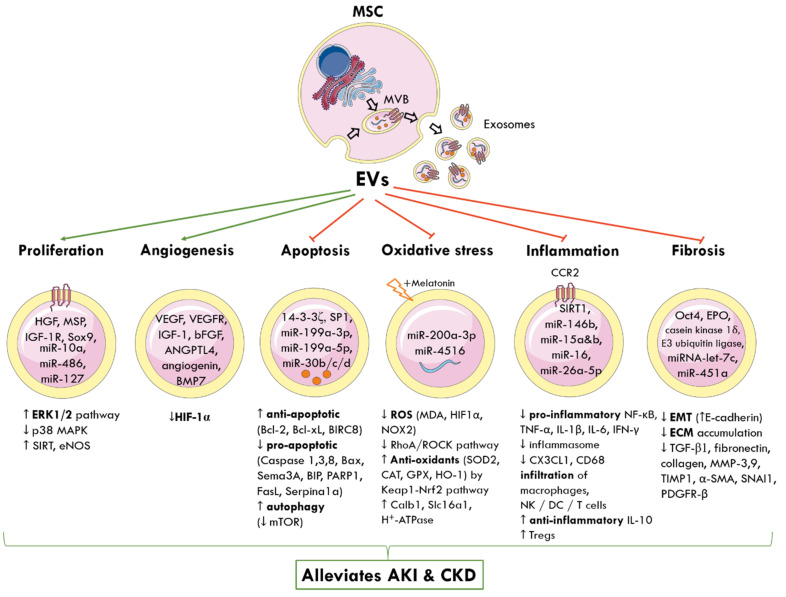
Molecular and signalling mechanisms by which MSC-derived EVs elicit nephroprotection following AKI and CKD. The cellular origin of one subtype of EVs, known as exosomes, from MVBs is shown. MSC-EVs deliver a cargo of miRNA, proteins, cytokines, and growth factors to nearby injured renal cells and alter gene expression. This reprograms pathophysiological pathways and promotes tubular proliferation and angiogenesis, and inhibits apoptosis, oxidative stress, inflammation, and fibrosis to alleviate AKI and CKD. Green arrows represent promotion; red arrows represent inhibition; up arrow (↑) indicates increased and down arrow (↓) indicates decreased levels or activity. MSC: mesenchymal stem cell; MVB: multivesicular bodies; EVs: extracellular vesicles; HGF: hepatocyte growth factor; MSP: macrophage-stimulating protein; IGF-1R: insulin growth factor-1 receptor; Sox9: SRY-box transcription factor; miR: miRNA; ERK: extracellular signal-regulated kinase; MAPK: mitogen-activated protein kinase; SIRT: sirtuin 1; eNOS: endothelial nitric oxide synthase; VEGF: vascular endothelial growth factor; VEGFR: VEGF-receptor; bFGF: basic fibroblast growth factor; ANGPTL4: angiopoietin-like 4; BMP7: basic morphogenetic protein 7; HIF-1α: hypoxia-inducible factor 1 alpha; SP1: proximal specificity protein 1; Bcl-2: B-cell lymphoma 2; Bcl-xL: B-cell lymphoma-extra-large; BIRC8: baculoviral IAP repeat containing 8; Bax: Bcl-2-associated X protein; Sema3A: semaphorin-3A; BIP: binding immunoglobulin protein; PARP1: poly [ADP-ribose] polymerase 1; Sepina1a: serpin family A member 1; mTOR: mammalian target of rapamycin; ROS: reactive oxygen species; MDA: malondialdehyde; NOX2: NADPH oxidase 2; RhoA: Ras homolog family member A, ROCK: Rho-associated protein kinase; SOD: superoxide dismutase; CAT: catalase; GPX: glutathione peroxidase; HO-1: haeme oxygenase 1; Keap1: Kelch-like ECH-associated protein 1; Nrf2: nuclear factor erythroid 2-related factor 2; Calb1: calbindin 1; Slc16a1: solute carrier family 16 member 1; ATP: adenosine triphosphate; CCR2: chemokine receptor type 2; NF-κB: nuclear factor kappa-light-chain enhancer of activated B cells; TNF-α: tumour necrosis factor alpha; IL-1β: interleukin-1β; CX3CL1: C-X3-C motif chemokine ligand 1; CD68: cluster of differentiation 68; NK: natural killer cells; DC: dendritic cells; Tregs: regulatory T cells; Oct4: octamer-binding transcription factor 4; EPO: erythropoietin; EMT: epithelial-to-mesenchymal transition; ECM: extracellular matrix; TGF-β1: transforming growth factor β1; MMP: matrix metalloproteinase; TIMP-1: tissue inhibitor matrix metalloproteinase 1; α-SMA: alpha smooth muscle actin; SNAI1: snail family transcriptional repressor 1; PDGFR-β: platelet-derived growth factor receptor β.

MSC-EVs can be engineered to carry different biomolecules and target the interplay of signalling pathways responsible for AKI and CKD (Figure 2).

### 9.1. mTOR

The mechanistic target of rapamycin (mTOR) is a nutrient-sensing protein kinase that suppresses autophagy and exacerbates podocyte damage in CKD, and its deactivation represents a target for MSC-exosomes [171,172]. Umbilical-MSC-exosomes increased expression of the autophagic marker protein, LC3B, and this inhibited phosphorylation of mTOR, thereby activating autophagy in cisplatin-triggered AKI [114] and STZ-induced T1DM [165]. Similarly, miR-486 from adipose-MSC-exosomes targeted Smad1 and this suppressed mTOR activity [169].

### 9.2. 14-3-3ζ

*14-3-3* is a family of adaptor proteins involved in regulating protein trafficking, cell cycling, signal transduction, and apoptosis, and it operates synergistically with mTOR to coordinate autophagy [173,174]. One isoform, 14-3-3ζ, offers protection from cell death due to hypoxia, chemotherapy, and growth factor deprivation [175]. Umbilical-MSC-exosomes, enriched with 14-3-3ζ, enhanced localisation of autophagy-related protein, 16L, to the outer surface of autophagosome precursors and this increased formation of autophagosomes [115]. By 14-3-3ζ inducing autophagy, renal cells were protected from apoptosis, cell proliferation increased, and this alleviated nephrotoxicity.

### 9.3. YAP

YAP is a transcription factor in the Hippo signalling pathway and co-localises with α-SMA in the nucleus of TECs to promote fibrosis through an unclear mechanism [176]. Umbilical-MSC-exosomes delivered casein kinase 1δ and E3 ubiquitin ligase β-transducin repeats-containing protein to trigger ubiquitination and degradation of YAP in TECs [166]. This reduced collagen and ECM deposition and attenuated fibrosis associated with UUO.

### 9.4. Oct-4

Oct-4 is known as one of the four transcription factors capable of reprogramming fibroblasts into induced pluripotent stem cells (iPSCs) [177] and it can downregulate Snail and the EMT [178]. Umbilical-MSC-EVs overexpressing Oct4 reduced apoptosis, promoted TEC proliferation, and rescued mice with IRI from fibrosis within two weeks [117].

### 9.5. SP1

MSC-EVs from human iPSCs can deliver sphinganine-1-phosphate 1 (SP1) to PTCs to directly bind the promoter region of sphingosine kinase 1 [122]. This increased SP1 expression and inhibited necroptosis in rats with IRI, elucidating a novel mechanism of EVs in nephroprotection.

### 9.6. Sox-9

Sox-9 is a transcription factor of the sex-determining region Y box family and may repair injured TECs [179]. Adipose-MSCs-exosomes upregulated Sox9 and prevented TGF-β1-induced transformation of TECs into a pro-fibrotic phenotype in mice with IRI [168]. Increased Sox9 stimulated TEC proliferation, attenuated AKI, and protected the development of tubulointerstitial fibrosis. Another study used two-photon microscopy to track human placenta-MSC-EVs migrating to kidneys injured by IRI. MSC-EVs promoted Sox9 activation in TECs to stimulate regeneration and reduce fibrosis within four weeks [142].

### 9.7. SIRT1

Sirtuin 1 (SIRT1) is an NAD^+^-dependent deacetylase of the sirtuin family that is expressed by various kidney cells during stress and inhibits inflammation, apoptosis, and fibrosis [180]. In sepsis-induced AKI, adipose-MSC-exosomes inhibited NF-κB-mediated transcription of pro-inflammatory cytokines in the SIRT1 pathway and reduced immune cell infiltration and apoptosis [141]. Furthermore, glial cell line-derived neurotrophic factor (GDNF) was transfected into adipose-MSCs, and their exosomes ameliorated fibrosis in mice with UUO [167]. This was mediated by SIRT1 signalling and its downstream target, phosphorylated endothelial nitric oxide synthase (p-eNOS), which activated endothelial function and angiogenesis and reduced PTC loss. Upregulation of SIRT3/eNOS by BM-MSC-EVs also improved angiogenesis and regeneration in cisplatin-triggered AKI [108].

### 9.8. MFG-E8

Milk fat globule-epidermal growth factor-factor 8 (MFG-E8) is a glycoprotein that inhibits the RhoA/ROCK signalling pathway. BM-MSC-EVs delivered MFG-E8 to rats with UUO and reduced inflammation, macrophage infiltration, mitochondrial damage, apoptosis, oxidative stress, and the EMT within two weeks [135].

### 9.9. Melatonin and PrP^c^

A recent study focused on the efficacy of melatonin in autologous MSC-based therapeutics for CKD [170]. Exposure of adipose-MSCs to melatonin upregulated expression of miR-4516 and cellular prion protein (PrP^C^), and “MT exosomes” were harvested. Adipose-MSCs were also collected from patients with CKD (CKD-MSCs) and incubated with MT exosomes, which promoted proliferation, mitochondrial activity, and angiogenic proteins, and protected cells from senescence. These MT exosome-treated CKD-MSCs improved neovascularisation and functional recovery when administered to mice with hindlimb ischaemia, which was mediated through miR-4516-PrP^c^ signalling.

## 10. Conclusions

MSCs have shown increasing potential in immunomodulation and regenerative medicine and their paracrine effects are mediated by the secretion of EVs [42,181,182,183,184,185]. MSC-EVs are advantageous over their counterpart whole cells due to a higher safety profile, lower immunogenicity, and the inability to directly form tumours [42,181,182,183,184,185]. The regenerative capacity of MSC-EVs is based on the cargo of biomolecules they deliver to injured renal cells, particularly the types of miRNA and ncRNA [60]. To minimise the level of reporting and publication bias in this review, multiple databases were searched, and two extensive tables were created to methodologically analyse 34 preclinical animal models of AKI and 26 of CKD. However, the heterogeneous nature of EVs means the extrapolated results are difficult to generalise. It can be concluded that MSC-EVs induce tubular proliferation, regeneration, and angiogenesis, and suppress apoptosis, oxidative stress, inflammation, the EMT, and tubulointerstitial fibrosis. By altering the pathogenesis of disease, MSC-EVs show promise in mitigating AKI and CKD and offering a novel therapeutic for patients.

There are some limitations of this review. There is no consensus regarding the reporting of studies using EVs as it is an emerging therapeutic and there is a lack of global standardisation in isolation, characterisation, and validation protocols [4,40,49]. Moreover, there are no established methods to differentiate the subtypes of EVs and therefore studies claiming to use a certain subtype cannot be verified and this makes comparison difficult. Additionally, there are functional differences in efficacy between EV subtypes. A recent study found adipose-MSC-MVs reduced proteinuria while only exosomes promoted natriuresis following chronic renal artery stenosis [147]. To minimise these effects, studies were compared based on the ISEV recommendations, according to the source of MSCs, EV subtype, protein content of administered EVs, and route of injection [49].

Furthermore, clinical translation is in its infancy and the conclusions are limited to preclinical animal models. They are monocausal and simplistic when compared to the multifactorial aetiologies of CKD and comorbidities, such as increasing age and cardiovascular disease, from which patients suffer [5]. Moreover, the selected animals are young and only a short duration of disease (weeks—months) is observed. Therefore, there is an increasing need for human clinical trials. In a phase II/III trial, twenty patients with Stage III and IV CKD (eGFR 15–60 mg/mL) received two doses of umbilical-MSC-EVs (100 μg/kg/dose) one week apart, and this increased eGFR and reduced serum creatinine, BUN, and urinary albumin creatinine ratio within one year [186]. The clinical improvement was attributed to increased anti-inflammatory cytokines and decreased TNF-α.

For translation of EV therapy to clinical practice, the following manufacturing issues surrounding optimal dosing, mode of injection, schedule of administration, potency assays, minimising dose toxicity, uniformity between batches, identification of EVs, and safety must be standardised [4,40,187]. This is inherently difficult when considering the heterogeneity of EVs, so each batch will display both donor and clone-specific differences [97,187]. Most studies used a single dose of EVs, but this may be insufficient to achieve a sustained effect in humans [113]. Multiple doses of EVs showed greater efficacy than single dosing but repeated injections decrease feasibility [98,109]. Most studies focused on intravenous injection but there is a shifting focus to delivering therapeutics to organs via their arterial blood supply. This maximises the efficacy at the target site while reducing its metabolism and systemic side effects [107]. The timing of EV injection is also significant whereby a recent study confirmed administration of BM-MSC-EVs after renal damage is more effective than delivering them prophylactically [154]. Most studies use in-house manufacturing and characterisation protocols to isolate EVs [97] and only a handful have published their adherence to good manufacturing practice criteria [187,188,189]. Further pharmaceutical regulation of the manufacture and delivery of EV-based therapeutics is required before they can be safely translated from the laboratory bench to the bedside [187].

In conclusion, MSC-EV therapy shows increasing potential for alleviating AKI and slowing the progression of CKD. Future studies should engineer the surface and cargo of EVs for superior specificity and develop optimal protocols for delivery and safe transition into clinical practice.

## Figures and Tables

**Table 1:** Comparison of methods to isolate exosomes from cell culture. RNA: ribonucleic acid; MSpec: mass spectrometry; SEC: size exclusion chromatography; dUC: differential ultracentrifugation; EVs: extracellular vesicles; PEG: polyethylene glycol.

Method	Differential Ultracentrifugation	Density Gradient	Size Exclusion Chromatography	Invitrogen Precipitation	Affinity-Based
**Principle**	Based on size and sedimentation rate by successive centrifugation at increasing speed and duration [61,62]	Based on density upon flotation or pelleting [45,63]	Based on separating sample molecules relative to pore size of chromatography gel column [57,64]	Compound polymer-based precipitation [63]	Affinity interaction between surface protein, sugar, or lipids, with antibodies coated on magnetic beads [48,57,63,65]
**Yield**	Intermediate	Low	Intermediate	High	Low
**Purity**	Low	Intermediate	High	Low	Highest
**Advantages**	- Most commonly used [56] - High sample capacity [63] - Protein and RNA components are unaffected - Suitable for MSpec, RNA sequencing	- High separation efficiency - Avoids crushing or deforming exosomes [57] - Reduces cellular and protein debris	- Size uniformity - Reduces protein contamination [66]	- Quick, simple [63] - Widely available [63] - Size uniformity - Suitable for RNA sequencing	- Short processing time [59] - Simple, convenient [59] - Unaffected by - exosome size - No expensive instrumentation [63] - Maintains exosome integrity [63]
**Disadvantages**	- Time-consuming [63] - Labour-intensive - Instrument-dependent: rotor type and centrifugal force [61,67] - Inappropriate for small sample volume [63] - Contaminating pellet of lipoproteins, nucleic acids [45,68]	- Time-consuming (density preparation) [63] - Instrument-dependent [63] - Must combine with SEC or dUC [57] - Not suitable for downstream MSpec	- Low extraction volume [66] - Extensive laboratory equipment [66] - Additional enrichment required [66]	- Precipitation of non-EVs, lipoproteins [69] - Expensive [63] - Affected by exosome diameter - Limited sample processing per reagent [63] - Must extract PEG before MSpec [57]	- Low sample capacity - Expensive reagents - Difficult elution of exosomes limits downstream applications [63]
